# Metagenomes from Coastal Marine Sediments Give Insights into the Ecological Role and Cellular Features of *Loki*- and *Thorarchaeota*

**DOI:** 10.1128/mBio.02039-19

**Published:** 2019-09-10

**Authors:** Lokeshwaran Manoharan, Jessica A. Kozlowski, Robert W. Murdoch, Frank E. Löffler, Filipa L. Sousa, Christa Schleper

**Affiliations:** a Archaea Biology and Ecogenomics Division, University of Vienna, Vienna, Austria; b Center for Environmental Biotechnology, University of Tennessee, Knoxville, Tennessee, USA; c Department of Microbiology, Department of Civil and Environmental Engineering, and Department of Biosystems Engineering and Soil Science, Knoxville, Tennessee, USA; University of Georgia; University of Waterloo; Pasteur Institute, Paris France

**Keywords:** *Archaea*, *Lokiarchaeota*, *Thorarchaeota*, ether lipids, eukaryotic evolution, reductive dehalogenase

## Abstract

The genomes of Asgard *Archaea*, a novel archaeal proposed superphylum, share an enriched repertoire of eukaryotic signature genes and thus promise to provide insights into early eukaryote evolution. However, the distribution, metabolisms, cellular structures, and ecology of the members within this superphylum are not well understood. Here we provide a meta-analysis of the environmental distribution of the Asgard archaea, based on available 16S rRNA gene sequences. Metagenome sequencing of samples from a salt-crusted lagoon on the Baja California Peninsula of Mexico allowed the assembly of a new *Thorarchaeota* and three *Lokiarchaeota* genomes. Comparative analyses of all known *Lokiarchaeota* and *Thorarchaeota* genomes revealed overlapping genome content, including central carbon metabolism. Members of both groups contained putative reductive dehalogenase genes, suggesting that these organisms might be able to metabolize halogenated organic compounds. Unlike the first report on *Lokiarchaeota*, we identified genes encoding glycerol-1-phosphate dehydrogenase in all *Loki*- and *Thorarchaeota* genomes, suggesting that these organisms are able to synthesize bona fide archaeal lipids with their characteristic glycerol stereochemistry.

## Abstract (precis)

Microorganisms of the superphylum Asgard *Archaea* are considered to be the closest living prokaryotic relatives of eukaryotes (including plants and animals) and thus promise to give insights into the early evolution of more complex life forms. However, very little is known about their biology as none of the organisms has yet been cultivated in the laboratory. Here we report on the ecological distribution of Asgard *Archaea* and on four newly sequenced genomes of the *Lokiarchaeota* and *Thorarchaeota* lineages that give insight into possible metabolic features that might eventually help to identify these enigmatic groups of archaea in the environment and to culture them.

## INTRODUCTION

Recent discoveries of novel microbial lineages through metagenomic analyses have led to a fundamentally refined perception of the diversity of both *Archaea* and *Bacteria* (1). Among these, *Lokiarchaeota* of the domain *Archaea* and related lineages, now collectively embraced in the superphylum Asgard *Archaea*, have garnered particular attention due to their close affiliation to eukaryotes (2, 3). Together with the finding that multiple Asgard genomes encode eukaryotic signature proteins (ESPs) (2, 3), these data have led to the assumption that relatives of Asgard archaea participated in the formation of the eukaryotic lineage. However, there are currently no cultured members of this archaeal phylum, making predictions about their biology and the functions of these ESPs in Asgard archaeal cell biology challenging. Currently, five major groups or phyla within the Asgard archaea have been defined based on phylogenetic reconstructions from metagenome assembled genomes (MAGs) obtained from a range of different environments, including *Lokiarchaeota* (3), *Thorarchaeota*, *Heimdallarchaeota*, *Odinarchaeota* (2), and, more recently, *Helarchaeota* (4).

While the positioning of the Asgard archaea in the tree of life is intriguing and the exact position of the emergence of the eukaryal line of descent is continuously being refined and sometimes debated (4–6), it is equally crucial to obtain more information on the ecological distribution and metabolisms of Asgard archaea. The characterization of the phylogenetic diversity within the superphylum and the environmental distribution of these organisms will inform future practices in detection. Also, understanding the particular environments Asgard archaea inhabit will advance knowledge of their roles in biogeochemical cycling and aid efforts to bring these intriguing organisms to culture. Diversity studies are important for proper analyses of Asgard archaeal abundance and distribution (for instance, in amplicon sequencing studies), for the design of specific primers aiding detection and enumeration with molecular techniques, and congruency between 16S rRNA gene and ribosomal protein trees to accurately define newly discovered members of the superphylum.

The first two publicly available *Lokiarchaeota* genomes were derived from the metagenomes of marine sediments at the Arctic Mid-Ocean Ridge and a freshwater aquifer (2, 3, 7). An early analysis of the specific geochemical parameters of the marine sediments in which *Lokiarchaeota*, (then known as the “Deep Sea Archaeal Group” [DSAG]) were found suggested a correlation with organic carbon and oxides of iron or manganese as potential electron acceptors (8). While a first study of the composite *Lokiarchaeota* GC14 genome suggested that these organisms were hydrogenotrophic autotrophs, fixing CO_2_ via the archaeal-type (tetrahydromethanopterin [THMPT]) Wood-Ljungdahl (WL) pathway (9), their genomes as well as that of all Asgard genomes indicate a potential for growth on organic compounds as well (10, 11).

*Thorarchaeota* genomes obtained from marine and estuary sediments (2, 12) as well as mangroves (13) appeared to be metabolically diverse with potential for degradation and uptake of peptides and carbohydrates as well as potential to fix dinitrogen and make selenoproteins (12, 13). Like *Lokiarchaeota*, the *Thorarchaeota* genomes contain genes for the THMPT-WL pathway, but in addition, the latter encode also a (bacterial-type) tetrahydrofolate (THF) version (13). Together with further observations indicating the potential for acetate or ethanol production, they were suggested to have a mixotrophic lifestyle. In addition to a complete Wood-Ljungdahl pathway a methyl-CoM reductase-like enzyme was recently found in *Helarchaeota*, similar to those detected in *Bathy*- and *Synthrophoarchaea* (4), which together with further observations indicated a potential for anaerobic oxidation of short-chain hydrocarbons in this group (4). So far, only members of the *Heimdallarchaeota* seem to have the potential of facultative aerobic growth, as they were recently found to encode a complete electron transport chain with terminal oxidase as well as the aerobic kynurenine pathway (14).

The present study updates the existing phylogeny within the Asgard archaea and includes a comprehensive analysis of their environmental distribution, with metadata on abiotic parameters such as pH and temperature ranges. This study also presents three *Lokiarchaeota* genomes and one *Thorarchaeota* genome from a hypersaline biomat from a salt lagoon close to Puertecitos, Baja California, Mexico, that was shown to harbor many newly identified archaea lacking cultured representatives (15). These genomes, together with those previously reported, help to compare genome contents between the *Lokiarchaeota* and the *Thorarchaeota* and to identify new metabolic capacities. The reported inventory includes putative reductive dehalogenase (RDase) genes in the genomes of *Loki*- and *Thorarchaeota* that might be instructive for cultivation attempts, and it sheds new light on lipid biosynthesis, as we identify genes for bona fide archaeal lipid biosynthesis in *Lokiarchaeota* that were previously missing in the assembled published genomes.

## RESULTS AND DISCUSSION

### Diversity and distribution of Asgard archaea.

**FIG 1:**
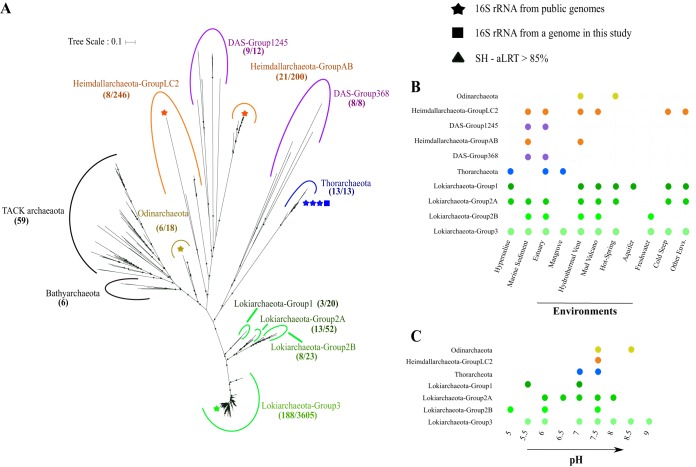
(A) 16S rRNA diversity of Asgard archaea based on representative gene sequences filtered from the SILVA database (v.132). Stars indicate full-length 16S rRNA gene sequences from publicly available genomes. Squares indicate full-length 16S rRNA gene sequences from genomes obtained in this study. In parentheses for each group is given the number of sequences used for calculating this refined phylogenetic tree (left) and the total number of sequences that we found being affiliated with it (right). Small triangles indicate bootstrap values of >85% (SH-aLRT). (B) Environmental distribution of Asgard archaea. (C) pH ranges of the environment from which genomes of Asgard group representatives have been found. Group representatives with a sequenced genome were recovered based on a literature survey.

In order to study the environmental distribution and diversity of known Asgard archaea, all affiliated 16S rRNA gene sequences were retrieved from the SILVA database (v.132). The initial phylogenetic analysis of 4,458 putative Asgard 16S rRNA gene sequences proved challenging at first, since no monophyletic groups were retrieved at the phylum level (see Fig. S1 in the supplemental material). However, conclusive results were obtained after excluding 388 potentially chimeric or low-pintail-quality sequences (see Table S4 posted at figshare [https://doi.org/10.6084/m9.figshare.9258947]). The remaining 4,070 sequences were filtered for a length greater than 1,400 bp, resulting in 2,857 unique Asgard sequences. After further clustering at a 99% identity threshold, 246 operational taxonomic units (OTUs) were obtained. In addition to these, 32 additional Asgard 16S rRNA gene sequences from available genomes (including one new full-length 16S rRNA gene obtained in this study) and other studies (16) that were not part of the SILVA database were included in phylogenetic reconstructions. The obtained backbone tree of the Asgard archaea (Fig. 1A; see Fig. S2 in the supplemental material) exhibited generally a similar clade organization for the *Lokiarchaeota* lineages as reported in earlier studies on smaller data sets (7, 8, 17). After mapping the remaining 1,213 shorter sequences to the reference sequences in this backbone tree, 229 sequences were found to have a different taxonomic affiliation than was reflected in our phylogenetic analyses (see Table S4 posted at figshare). For example, there were 36 sequences that had a different taxonomic affiliation in the SILVA database (v.132) than here with the refined phylogeny, and moreover, 193 of the good-quality shorter sequences did not match any of the reference sequences in the refined phylogeny with the given threshold (see Table S4 posted at figshare). One of two distinct groups of sequences earlier assigned as the DAS (domain archaeal sequences) group (16) was found to be a sister group of the *Thorarchaeota* clade, and the second branched in between two *Heimdallarchaeota* clades. Further inferences regarding the phylogeny and naming of these groups should await full genome sequence analyses.

10.1128/mBio.02039-19.1 FIG S1 Unrooted phylogenetic tree (ML) of the 16S rRNA gene sequences (nonredundant, representatives clustered at 99% identity threshold) from the Asgard *Archaea* superphylum obtained from SILVA (v.132). Similar to Fig. 1, they were aligned with the well-curated full-length 16S rRNA gene sequences from the TACK archaea. The colors represent the taxonomic affiliation of these sequences in the SILVA database. Note there are no *Thorarchaeota* 16S rRNA gene sequences available in the SILVA database. The *Thorarchaeota* sequences were full-length sequences from available genomes of previous studies (2, 12, 13, 16) and from our study. Download FIG S1, PDF file, 0.2 MB. Copyright © 2019 Manoharan et al. 2019 Manoharan et al. This content is distributed under the terms of the Creative Commons Attribution 4.0 International license.

10.1128/mBio.02039-19.2 FIG S2 Rooted version of the refined phylogeny (Fig. 1). The TACK and *Bathyarchaeota* sequences were used as an outgroup to root this tree. The different colors represent the different Asgard phyla. The 16S sequences from genomes are represented in boldface. The triangles represent the branch support: SH-aLRT value of >85%. Download FIG S2, PDF file, 0.07 MB. Copyright © 2019 Manoharan et al. 2019 Manoharan et al. This content is distributed under the terms of the Creative Commons Attribution 4.0 International license.

A thorough literature survey of metadata identified *Lokiarchaeota* as the group with the widest occurrence in different ecosystems compared to the other Asgard groups (Fig. 1B). However, out of the 4,070 16S rRNA gene sequences used in this analysis, environmental information was only available for 531 (13%). Of these, 22% (117) matched to one particular *Lokiarchaeota* OTU (group 3) found in hypersaline environments. A previous study reported that 60% of all *Lokiarchaeota* (then termed DSAG) sequences available at the time in the SILVA database (v.104) originated from hypersaline ponds in Guerrero Negro (Baja California, Mexico) (8). *Lokiarchaeota* are also found in the widest pH range (5 to 9) of any Asgard archaea, whereas *Odinarchaeota* were only found in neutral to moderately alkaline (pH 7.5 to 8.5) environments (Fig. 1C). However, the picture of the environmental distribution of Asgard lineages might change when more sequence data become available that are specifically dedicated to the detection of this superphylum in various environmental microbiomes.

### Novel genomes of *Loki*- and *Thorarchaeota*.

**FIG 2:**
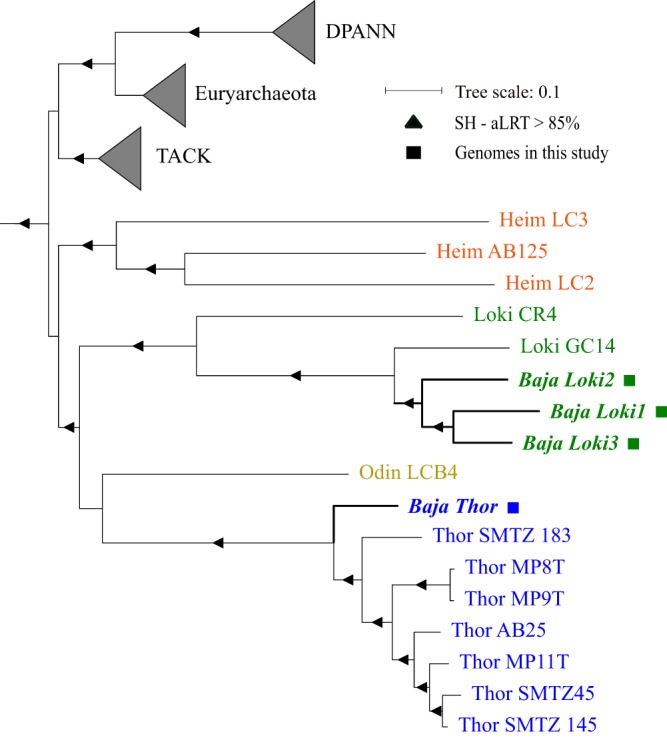
Concatenated ribosomal protein tree of Asgard archaea (blue, *Thorarchaeota*; yellow, *Odinarchaeota*; green, *Lokiarchaeota*; and red, *Heimdallarchaeota*). Selected representative archaeal genomes from other phyla are shown in black. The maximum likelihood phylogeny was reconstructed in IQ-TREE with the LG+F+I+G4 model. The alignment was trimmed to 6,732 positions with the BMGE (Block Mapping and Gathering with Entropy) tool. Branch support is denoted by triangles with an SH-aLRT value of >85%.

We were able to reaffirm the presence of *Lokiarchaeota* (DSAG) in hypersaline environments by a diversity study of the prokaryotic community in another hypersaline environment in the Baja California region, a closed salt lagoon near Puertecitos, Mexico (15). Here, we used differential coverage binning from a metagenomic analysis of one of the sediment samples from that location (1-cm depth) and were able to acquire four new metagenome assembled genomes (MAGs) of Asgard archaea. One of these was of high quality and affiliated with the *Thorarchaeota*, while three additional MAGs were of medium quality and affiliated with *Lokiarchaeota* (according to MAG standards) (18) (Table 1). The *Thorachaeota* genome from this study (assigned as “Baja_Thor”) was the most complete of all Asgard genomes available in the database and had the least contamination according to CheckM analysis. The taxonomic affiliation of all four genomic bins that was based on their single-copy protein matches to the NR database was confirmed with the phylogenetic analysis of concatenated ribosomal proteins (Fig. 2). Published *Lokiarchaeota* genomes are generally larger than the available *Thorarchaeota* genomes, a trend that was also observed in this study, with the *Thorarchaeota* (Baja_Thor) genome size of 3.1 Mb notably smaller than the *Lokiarchaeota* genomes, which ranged from 3.9 to 4.1 Mb in size. The Orthologous Average Nucleotide Identity (OrthoANI) (19) values for the Baja_Thor genome compared with other *Thorarchaeota* genomes ranged from 65.6% to 66.2%. Individually, the *Thorarchaeota* genome from this study (Baja_Thor) was most closely related to the Thor_MP9T genome obtained from mangroves (13). The three *Lokiarchaeota* genomes obtained in this study were more closely related to each other than the *Lokiarchaeota* genomes previously published (2), with their OrthoANI values ranging from 66.8% to 68.2%.

**TABLE 1:** Characteristics of the Asgard-affiliated MAGs obtained from a metagenomic data set from the upper layer (top 1 cm) of a salt lagoon in Baja California

Parameter	Result for Bin_id:			
	Baja_Thor	Baja_Loki1	Baja_Loki2	Baja_Loki3
Completeness, %	92.99	75.19	83.64	88.32
Contamination, %	3.74	1.4	9.35	3.74
Strain heterogeneity	0	0	0	0
Size, Mb	3.1	3.9	3.8	4.1
Scaffolds, no.	19	274	114	137
GC, %	41.2	33.2	32.6	33.6
*N*_50_, bp	548,263	20,187	44,367	42,570
Length, bp				
Max	668,889	69,370	119,378	143,576
Mean	166,420.1	14,312.4	33,109.3	30,262.1
Coverage, X	167.8	15.77	9.21	7.13
Proteins, no.	2,909	3,425	3,434	3,918
Coding density, %	90.10	82.80	85.20	86.50
Reads mapping to genome, %	2.07	0.27	0.15	0.14

### Pangenomic analysis of *Loki*- and *Thorarchaeota*.

**FIG 3:**
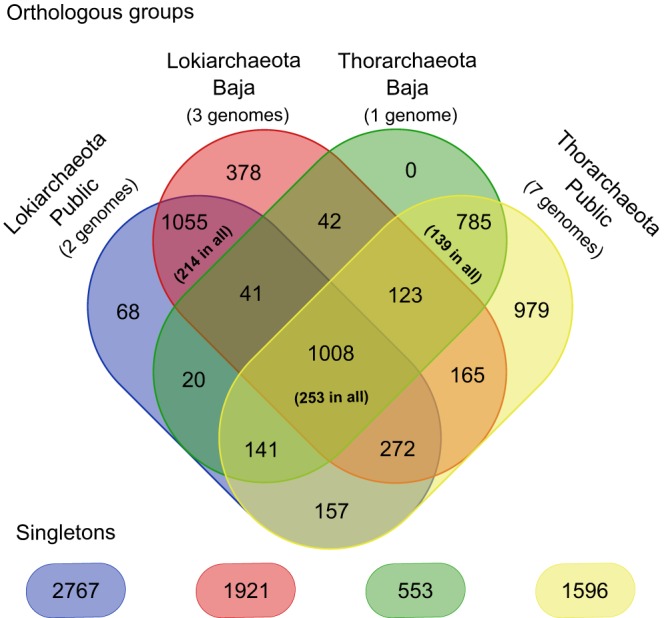
Shared and specific *Loki*- and *Thorarchaeota* core genomes. The Venn diagram represents the shared orthogroups between the public *Loki*- and *Thorarchaeota* genomes together with the four genomes obtained from Baja California. Numbers in parentheses represent the number of group-specific orthogroups present in all *Lokiarchaeota* (5 genomes) or all *Thorarchaeota* (8 genomes). Numbers in the center indicate orthogroups present in all *Loki*- and *Thorarchaeota* (13 genomes [shared core genome]).

Since a number of genomic similarities between *Lokiarchaeota* and *Thorachaeota* had been pointed out earlier and since they often share the same environment and can even be found in the same sample (as in our study), we were interested in performing a general pangenomic comparison. A pairwise all-versus-all BLAST and clustering analysis was therefore performed in Orthofinder (20) with default parameters. Among the 47,666 proteins present in the 13 MAGs used for comparative analysis, 86% (40,712) belonged to one of the 5,234 protein clusters containing sequences from at least two genomes (Fig. 3). The remaining 6,954 sequences were classified as singletons. Using a strict criterion of presence in all genomes, the core Loki-Thor genome (i.e., protein families with at least one protein in all of the 13 genomes) consists of 253 clusters (∼5% of total protein families), in which the most-represented functional categories belong to information storage and processing related to translation (49 clusters) followed by energy metabolism (37 clusters). The *Lokiarchaeota*-specific clusters represented ∼29% (1,501) of the sequences, in which 214 protein clusters were present in all *Lokiarchaeota* and thus can be considered to be encoded by the *Lokiarchaeota* core genome. The *Thorarchaeota*-specific clusters were represented by ∼34% of the sequences (1,764), with 139 clusters present in all *Thorarchaeota* genomes. The remaining 1,969 clusters had members from both taxonomic groups.

**FIG 4:**
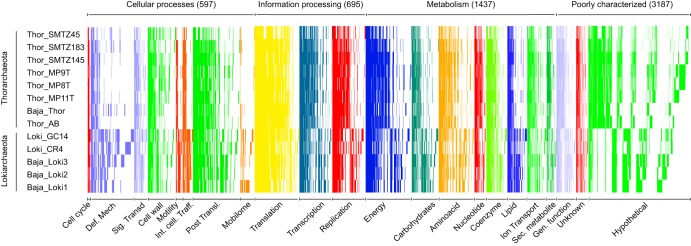
Presence/absence matrix of the different orthogroups in the different *Loki*- and *Thorarchaeota* genomes. The orthologous groups are sorted for each arCOG class from present in all 13 genomes to singletons (present in 1 genome). For better visualization, singletons belonging to the hypothetical protein ArCOG are not shown.

Based on arCOG annotations, significant differences between the *Loki*- and the *Thorarchaeota* genomes became evident (Fig. 4). However, most of these differences corresponded to clusters lacking functional annotations or representing hypothetical proteins. Clear differences between the two groups were mostly observed in protein clusters related to mobile elements and defense mechanisms. Whereas *Lokiarchaeota*-specific protein clusters were mostly affiliated with transposable elements and recombinases (35/41 protein clusters), *Thorarchaeota*-specific protein clusters contained phage terminases and multidrug transport systems (11/15 protein clusters) (see Table S5 posted at figshare [https://doi.org/10.6084/m9.figshare.9258968]). The protein clusters for carbon metabolism (of both *Thor*- and *Lokiarchaeota*) contained at least one gene from all enzymatic complexes responsible for carrying out the steps from the archaeal version of the Wood-Ljungdahl (WL) pathway, as well as many of the enzymes for the bacterial (folate-dependent) WL pathway. However, the formate dehydrogenase enzyme responsible for the initial step of the reduction and fixation of CO_2_ through the bacterial WL-THF pathway was not found in any of the *Loki*- and *Thorarchaeota* genomes. Interestingly, even if several of the CO dehydrogenase (*cdh*) subunits were found encoded in genomes from the two groups, in the Baja_Thor genome, which is the most complete among all genomes, no *cdh* subunits were found, although genes encoding other enzymes of the THMPT-WL pathway were present. Recent analysis of the WL pathway indicated its great modularity and flexibility in archaea, in terms of both evolution as well as functional diversity (21). Consistent with previous findings (13), an incomplete tricarboxylic acid (TCA) cycle was found (see Table S6 posted at figshare [https://doi.org/10.6084/m9.figshare.9259094]) in all *Loki*- and *Thorarchaeota* analyzed, as well as potential pathways for degradation and assimilation of proteins. The latter included enzymes coding for extracellular proteases and peptidases: e.g., aminopeptidases like *pepDPFN* and serine proteases like *aprE* (see Table S6 posted at figshare). In addition, genes encoding an arsenic efflux pathway and selenocysteine biosynthesis (13) (see Table S6 posted at figshare) were present in all genomes from both groups. Similarly, several membrane transporter genes, including those encoding the enzymes for multisugar compounds (see Table S6 posted at figshare), like MFS family permease genes, were also found in all genomes analyzed.

### Putative reductive dehalogenases.

Halogenated organic compounds have been introduced into the environment through anthropogenic activities but also occur naturally, particularly in marine sediments (22). Interestingly, from the orthogroup analysis, we identified genes encoding putative reductive dehalogenases (RDases) in nearly all *Thor*- and *Lokiarchaeota* genomes (see Fig. S3 in the supplemental material). The three genomes lacking RDase genes were least complete (Loki_CR4, Baja_Loki1, and Thor_SMTZ_45), providing an incomplete picture of gene content (see Table S3 posted at figshare [https://doi.org/10.6084/m9.figshare.9258926]). The *Lokiarchaeota* GC14 genome has two copies of a putative RDase gene (86.8% amino acid identity); however, this genome may be a composite of two genomes for closely related *Lokiarchaeota*, and it is not clear if a single genome harbors two putative RDase genes (3). To date, Ferroglobus placidus is the only other archaeon whose genome encodes a putative RDase (23).

10.1128/mBio.02039-19.3 FIG S3 Maximum likelihood tree of the archaeal RDases (indicated by colored names and branch lines), orthologous groups of 4Fe-4S ferredoxin and RDase proteins as defined by the EggNOG 4.5.1 database, and representative epoxyqueosine reductase (QueG) sequences derived from KEGG ortholog family K18979. The enlargement displays the taxonomic origins and names of the most closely related proteins in the EggNOG 4.5.1 database. Bootstrap values are indicated at the nodes. Download FIG S3, PDF file, 0.10 MB. Copyright © 2019 Manoharan et al. 2019 Manoharan et al. This content is distributed under the terms of the Creative Commons Attribution 4.0 International license.

**FIG 5:**
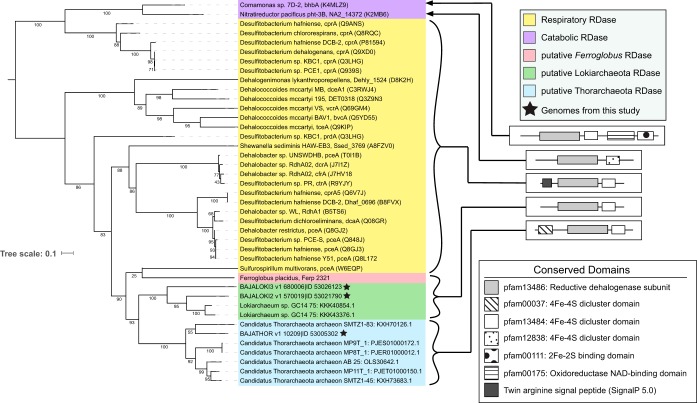
Maximum likelihood tree of the putative archaeal RDases and RDases with demonstrated activity (75). UniProt accession numbers are included following the species designation and the gene name. Bootstrap values are indicated at the nodes. Consensus conserved domain organizations among indicated groups are shown on the right as determined by NCBI CD search for pfam domains and SignalP 5.0 for TAT signals.

The known and well-studied RDases contain a conserved RDase domain, pfam13486. The domain organizations of RDases are variable and fall into at least two categories. The RDases involved in organohalide respiration contain a C-terminal 4Fe-4S dicluster binding domain (pfam13484) or alternatively a 4Fe-4S dicluster domain (pfam12838) and an N-terminal twin arginine translocation (TAT) signal sequence (Fig. 5) (24). There are alternative domain organizations associated with catabolic versus respiratory RDases, which may include an additional flavin adenine dinucleotide (FAD)- or NAD-binding and 2Fe-2S-binding domains at the C termini (25). All of the putative RDases identified among the Asgard archaea share core RDase domains, including reductive dehalogenase (pfam13486), and the 4Fe-4S dicluster binding domains (pfam13484) (Fig. 5; see Fig. S4 in the supplemental material). The thorarchaeotal sequences, on the other hand, contain an additional 4Fe-4S domain (pfam00037) near the N terminus, a domain organization that has neither been observed neither among proven-function RDases (Fig. 5) nor by data mining and characterization of RDases in public databases (26). A TAT signal sequence responsible for the translocation of the enzyme across the membrane is present in all experimentally verified respiratory RDases (27) but is absent in catabolic RDases studied to date (25). TAT signals are not found in any of the *Thor*- or *Lokiarchaeota* genome sequences (Fig. 5; Fig. S4), suggesting that these putative RDases are not involved in a respiratory process; however, since archaeal signal peptides are less characterized, their absence does not prove that translocation across the cytoplasmic membrane does not occur.

10.1128/mBio.02039-19.4 FIG S4 Alignment of the putative RDase proteins from all *Loki*- and *Thorarchaeota* together with representatives of characterized RDases (75). The amino acid residues explained in the crystal structure of PceA interacting with the different cofactors and the substrate (29, 30) are defined as follows: vcrA_Dmc, vinyl chloride RDase from Dehalococcoides mccartyi; tceA_Dmc, trichloroethene RDase from Dehalococcoides mccartyi; bvcA_Dmc, vinyl chloride RDase from Dehalococcoides mccartyi; cprA_Ddh, ortho-chlorophenol RDase from Desulfitobacterium dehalogenans; prdA_Dsp, tetrachloroethene RDase from *Desulfitobacterium* sp. KBC1; rdhA_Dha, RDase from Desulfitobacterium hafniense; pceA_Dha, tetrachloroethene RDase from Desulfitobacterium hafniense; cprA_Dch, ortho-chlorophenol RDase from Desulfitobacterium chlororespirans; cprA_Dha, ortho-chlorophenol RDase from Desulfitobacterium hafniense; pceA_Smu, tetrachloroethene RDase from Sulfurospirillum multivorans. Download FIG S4, PDF file, 0.1 MB. Copyright © 2019 Manoharan et al. 2019 Manoharan et al. This content is distributed under the terms of the Creative Commons Attribution 4.0 International license.

A phylogenetic analysis comparing the Asgard archaeal putative RDases with the most closely related ortholog groups in the EggNOG database (28) revealed that the enzymes encoded on the *Thor*-, *Lokiarchaeota*, and Ferroglobus placidus genomes cluster among bacterial RDases rather than the most closely related groups of archaeal proteins (Fig. S3). Notably, the putative Asgard RDases cluster together, but separately from the RDase found in *F. placidus*. The most closely related functionally characterized enzyme (∼30 to 33% amino acid identity) is PceA from Sulfurospirillum multivorans. The PceA RDase is involved in organohalide respiration with tetrachloroethene as electron acceptor and reductive dechlorination of other organohalogens, including halogenated phenolic compounds, has been demonstrated (29, 30). Three highly conserved amino acid residues (Tyr^246^, Arg^305^, and Asn^272^) in the active site of PceA (30) are conserved in the putative RDases from *Loki*- and *Thorarchaeota* (Fig. S4). Taken together, these data suggest that the putative Asgard RDases probably represent enzymes with RDase function that may have been acquired by horizontal gene transfer from bacteria.

The gene neighborhoods of the putative Asgard RDase genes are notably conserved within the *Thorarchaeota* and within the *Lokiarchaeota*, but not between the two groups (see Fig. S5 in the supplemental material). The observed conservation of synteny suggests that the metabolic roles of these RDases may be informed by the functions encoded by consistently present surrounding genes (31). This is in contrast to the case of previously described respiratory and catabolic RDases, which show inconsistent gene neighborhoods. While a handful of genes speculated to be involved in functions such as molecular chaperoning, electron transfer, and corrinoid scavenging are conserved in some RDase-containing lineages, the only gene consistently associated with respiratory RDase genes encodes an accessory B protein, which tethers the RDase to the membrane, thereby enabling its role in a respiratory process. This pattern is nearly ubiquitous in respiratory RDases (reviewed in reference 24), although a subset of putative RDase genes appears to have fused with the respective gene encoding the accessory B protein (3, 31a). Genes encoding B proteins were not detected in *Thorarchaeota* and *Lokiarchaeota* genomes, nor were any RDase gene fusions detected. Many of the syntenic genes neighboring the archaeal RDases can be functionally annotated. In the *Lokiarchaeota*, the putative RDase is found near a trio of conserved genes annotated as arginyl-tRNA synthase (K01480), agmatinase (K01480), and deoxyhypusine synthase (K00809), suggesting a possible role in metabolism of small organoamines. These three syntenic genes encode proteins with ∼75% amino acid identity between the Baja_Loki3 and Loki_GC14 genomes (Fig. S5A). In the *Thorarchaeota*, the genes surrounding the putative RDase genes are remarkably syntenic (Fig. S5B), although their biological function is difficult to infer and a considerable synteny is not specific to this region but found along the whole *Thorarchaeota* genomes.

10.1128/mBio.02039-19.5 FIG S5 Synteny analysis of gene neighborhoods of the putative RDase-encoding genes from *Lokiarchaeota* (A) and *Thorarchaeota* (B) genomes. Shaded gray bars between genes represent BLASTP similarities between putative gene products with e < 0.00001. Genes conserved across at least three gene neighborhoods and can be functionally annotated by KEGG, COG, or TIGRFAM are indicated by colored bars. Download FIG S5, PDF file, 0.2 MB. Copyright © 2019 Manoharan et al. 2019 Manoharan et al. This content is distributed under the terms of the Creative Commons Attribution 4.0 International license.

The analyzed genomes did not seem to have complete pathways for corrinoid biosynthesis. However, a cobalamin transporter (*cbiM*), which is necessary for the function of this enzyme (25, 32), is also present in both *Loki*- and *Thorarchaeota* genomes (see Table S6 posted at figshare [https://doi.org/10.6084/m9.figshare.9259094]). Orthogroup and BLAST analyses revealed that the Baja *Loki*- and *Thorarchaeota* genomes also contain potentially complete versions of pathways for aromatic amino acid degradation, similar to what has been found in Ferroglobus placidus (33) (see Table S7 posted at figshare [https://doi.org/10.6084/m9.figshare.9259097]). In particular, genes encoding the key enzyme phenylacetyl-coenzyme A (CoA) ligase present in the Baja_Loki3 genome share sequence similarities resulting in up to 52% amino acid identity with the enzyme of *F. placidus* (33, 34). The degradation of tyrosine includes 4-hydroxyphenylacetate as an intermediate, and given the presence of genes encoding this pathway, both *Loki*- and *Thorarchaeota* might dechlorinate chlorinated aromatic compounds, including aromatic amino acid derivatives, using an RDase and then channel dechlorination products into this pathway, leading directly to C and N scavenging from aromatic amino acids. The enzyme indolepyruvate:ferredoxin oxidoreductase (*Ior*), which is characterized to be involved in peptide fermentation through oxidative decarboxylation of pyruvate in archaea (35, 36), was also identified to be encoded within *Loki*- and *Thorarchaeota* genomes (see Table S7 posted at figshare). In several methanogens, however, the phenylacetyl-CoA ligase and *Ior* are also present, being part of one of the three aromatic amino acid biosynthetic pathways (or of pABA) and indicating assimilation of indoleacetate and phenylacetate by reductive carboxylation (37, 38). Thus, the genomic identification of this pathway within *Loki*- and *Thorarchaeota* genomes *per se* is not a proof of its physiological function. In any case, the utilization of chlorinated amino acid derivatives, which have been observed as natural products (39), would give *Loki*- and *Thorarchaeota* a competitive advantage in oligotrophic environments.

### Lipid biosynthesis pathways.

**FIG 6:**
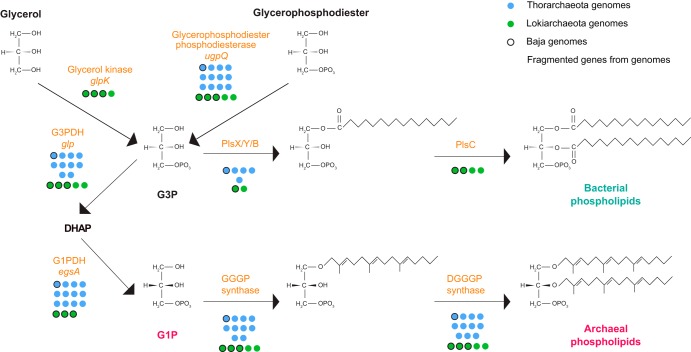
Lipid membrane biosynthesis predictions from all *Loki*- and *Thorarchaeota* genomes. The colored circles (green, *Lokiarchaeota*; blue, *Thorachaeota*) represent the presence or absence of the enzymes in the pathway. Colored circles with a black border represent the genomes from this study. The colored semicircle represents the predicted putative fragment of the G1PDH enzyme in *Lokiarchaeota* GC14. The predictions and pathways are based on a prior publication (40). G1P, glycerol-1-phosphate; G3P, glycerol-3-phosphate; DHAP, dihydroxyacetone phosphate; GGGP, geranylgeranylglyceryl phosphate; and DGGGP, digeranylgeranylglyceryl phosphate.

The so-called “lipid divide” (i.e., the presence of fundamentally different lipids in the archaea as opposed to lipids in bacteria and eukaryotes) has long been a topic of interest when discussing transitions in early evolution and in particular the origin of the eukaryotic cell (40). Considering the close affiliation of Asgard archaea with eukaryotes, it is particularly interesting to study the archaeal lipids (2, 3). Intriguingly, an archaeal glycerol-1-phosphate dehydrogenase (G1PDH) was found in neither the first analysis of the *Lokiarchaeota* genome GC14 nor the Loki_CR4 genome (3, 40). This enzyme determines the stereo-configuration of archaeal lipids and is a hallmark enzyme of archaea. However, it was also found to be missing in the genomes of marine group II and III archaea, and a homolog has recently been found in a bacterium, Bacillus subtilis (40). Interestingly, the protein from Bacillus subtilis has been biochemically characterized and was shown to produce G1P for archaeal-type phosphoglycerolipid synthesis in bacteria (41, 42). Our study of the extensive orthologous group analysis revealed that all *Thorarchaeota* genomes and all three *Lokiarchaeota* genomes from Baja California possess a G1PDH (Fig. 6; see Fig. S6 and Fig. S7 in the supplemental material), and we identified a truncated G1PDH in *Lokiarchaeota* GC14 located at the end of a scaffold, which may have complicated previous annotation attempts. In addition, we identified a G1PDH homolog in the most complete of the recently newly released *Lokiarchaeota* genomes (14). The sequence alignment (Fig. S6) confirmed similarity to known G1PDH and showed conservation in residues, including Asp^168^, His^248^, and His^264^, for binding of Zn^2+^ ions in the G1PDH enzymes of *Loki*- and *Thorarchaeota*, identical to different G1PDH representatives (41). Residues for NAD(P)H binding in the G1PDH were also conserved in *Thorarchaeota* (6/6 conserved) and mostly in *Lokiarchaeota* (4/6). However, a difference was seen in the residues of the G1PDH for binding of the dihydroxyacetone phosphate (DHAP) moiety, as these were well conserved in the *Thorarchaeota* but not in the *Lokiarchaeota* (Fig. S6). Phylogenetic analysis revealed that the enzymes were part of two basal branches, with *Thorarchaeota* forming a monophyletic lineage with archaea and *Lokiarchaeota* grouping with enzymes from *Bacillus* spp. and *Geobacillus* spp. (Fig. S7) (41, 43). Although phylogenetic analyses and conserved amino acid residues suggest these enzymes are in fact G1PDHs, biochemical characterizations will be necessary to provide more support.

10.1128/mBio.02039-19.6 FIG S6 Alignment of the predicted G1PDH (glycerol-1-phosphate dehydrogenase) from the *Loki*- and *Thorarchaeota* genomes together with the G1PDH (*araM*) from Bacillus subtilis and other representative archaeal G1PDH (*egsA*). The substrate, coenzyme, and metal binding sites are as described for *egsA* (41, 43). Arc_fu, Archaeoglobus fulgidus; Pyr_ab, Pyrococcus abyssi; Aer_pe, Aeropyrum pernix; Met_th, Methanococcus thermoautotrophicus; Sul_so, Sulfolobus solfataricus. Download FIG S6, PDF file, 0.09 MB. Copyright © 2019 Manoharan et al. 2019 Manoharan et al. This content is distributed under the terms of the Creative Commons Attribution 4.0 International license.

10.1128/mBio.02039-19.7 FIG S7 Unrooted phylogenetic tree (ML) of representatives from glycerol-1-phosphate dehydrogenases (G1PDH) and glycerol-3-phosphate dehydrogenases (G3PDH) that were included in the previous study on lipid biosynthesis in archaea and bacteria (40). The putative G1PDH fragment from the *Lokiarchaeota* GC14 genome was removed from this tree, due to its short protein sequence. The circles represent the branch support (>85) calculated by SH-aLRT. Download FIG S7, PDF file, 0.04 MB. Copyright © 2019 Manoharan et al. 2019 Manoharan et al. This content is distributed under the terms of the Creative Commons Attribution 4.0 International license.

No *Loki*- or *Thorarchaeota* genome had a glycerol-3-phosphate dehydrogenase (G3PDH/*gps*) involved in forming G3P from DHAP, as needed for the formation of bacterial lipids. Although all genomes seemed to contain several homologs of glycerol kinase (*glpK*), a detailed phylogenetic analysis revealed that most were homologs of bacterial carbohydrate kinases. Only four genes from *Lokiarchaeota* genomes represented homologs of the bona fide glycerol kinase (see Fig. S8 in the supplemental material). Consequently, G3P could in principle represent the backbone for bacterial-/eukaryal-like lipids in these *Lokiarchaeota* (as speculated earlier [40]); however, the presence of a G3PDH/*glpAD* allows them to further metabolize G3P into DHAP like other heterotrophic archaea (Fig. 6; see Table S8 posted at figshare [https://doi.org/10.6084/m9.figshare.9259103]) (40). Alternatively, G3P could also be synthesized from glycerol phosphodiester via the diesterase UgpQ, which was found in all *Loki*- and *Thorarchaeota* genomes. While all *Loki*- and most *Thorarchaeota* genomes contained genes for connecting the isoprenoid side chains to the glycerol backbone via ether linkage (GGGP and DGGP synthases), they also contained genes for enzymes forming ester bonds to a fatty acid (FA) mimicking the bacterial synthesis. Such enzymes include homologs of acyl transferases, i.e., PlsY involved in ester-linking a fatty acid to a G3P in the *sn*-1 position (44), which we found in *Loki*- and *Thorarchaeota* genomes and could not be detected in any other archaea, as well as PlsC (responsible for ester-linking FA to G3P in the *sn*-2 position) found in four *Lokiarchaeota* genomes and also in few other archaea.

10.1128/mBio.02039-19.8 FIG S8 Phylogenetic tree (ML) of the predicted glycerol kinase (*glpK*) proteins from *Lokiarchaeota* (green) shown together with the other archaeal glycerol kinases (yellow), with carbohydrate kinases as the outgroup (blue). This phylogenetic tree was reconstructed based on the glycerol kinase sequences reported previously (40). Both SH-aLRT (left) and the ultrafast-bootstrap values (right) are shown in each of the nodes. Download FIG S8, PDF file, 0.3 MB. Copyright © 2019 Manoharan et al. 2019 Manoharan et al. This content is distributed under the terms of the Creative Commons Attribution 4.0 International license.

Taken together, our findings show that *Loki*- and *Thorarchaeota* have all the genes necessary for the synthesis of bona fide archaeal lipids. In addition some *Lokiarchaea*, but rather not *Thorarchaeota*, might potentially be able to produce bacterial-type lipids, but the distribution of the respective genes (*glpK*, *plsY*, and *plsC*) is scattered. Since genes for the archaeal and bacterial lipid biosynthesis pathways are present in *Loki*- and *Thorarchaeota*, and since genes for the synthesis of fatty acids have also been proposed (45), we can also not rule out the possibility that chimeric lipids could be produced—for example, consisting of an isoprenoid chain ester bonded to a G3P—as has been suggested before (45). This observation strengthens claims of previous studies, including the first Lokiarchaeum composite genome (Lokiarchaeum_GC14), which proposed that *Lokiarchaeota* could use a hypothetical DGGGP synthase to form ether-linked isoprenoids with G3P stereochemistry, use PlsC to form ester-linked fatty acids with G3P chemistry, or use PlsC in a hypothetical function to produce chimeric membranes with ether-linked isoprenoids in the *sn*-1 position and ester-linked fatty acids in the *sn*-2 position of a G3P backbone (40). The additions of the Baja_Loki and Baja_Thor genomes might support the idea that these Asgard archaea could also produce both types of membranes or a heterochiral membrane. A recent study on lipid engineering showed that biochemically the GGGP synthase enzyme of the archaeon Methanococcus maripaludis, responsible for linking an isoprenoid chain GGPP to G1P, had only a slightly higher preference for G1P over G3P and that, in practice, a mixed heterochiral membrane made an Escherichia coli strain more robust to cellular stressors (40, 46).

### Conclusions.

A range of metabolic capacities points to potentially versatile metabolisms in *Loki*- and *Thorarchaeota*. The finding of putative reductive dehalogenase genes in the genomes of both groups (see also reference 10) together with potential genes encoding enzymes for aromatic amino acid utilization points to an adaptation of the organisms to specific niches. In the salt lagoon from which the genomes reported here were obtained, there might exist a pool of chlorinated compounds arising from the breakdown of organic material followed by subsequent chlorination, through biotic (47) or abiotic (47, 48) reactions, making these substrates inaccessible to nondehalogenating organisms and providing a competitive advantage for organisms with the ability to dehalogenate. Our study expands previous work (2, 7, 8, 10, 14) and shows that *Loki*- and *Thorarchaeota* are commonly found in anoxic environments, where they are involved in nutrient cycling potentially including halogenated compounds. This finding might be instructive for selective enrichments of the organisms from marine sediments or other locations. Further, our data provide evidence that *Lokiarchaeota*, like other Asgard archaea, can produce glycerol-1-phosphate for bona fide archaeal phosphoglycerolipids, which might as well assist in tracing them in environmental samples, albeit confirmations can only be obtained through cultivation of the organisms.

## MATERIALS AND METHODS

### Asgard 16S rRNA gene diversity.

The reference sequences of the 16S rRNA genes that belong the Asgard *Archaea* superphylum were downloaded from the latest SILVA rRNA database (v.132; 20 December 2017). The following filtering steps were implemented to these downloaded sequences to make a better reference phylogeny and taxonomic annotation of Asgard archaeal 16S rRNA gene sequences. (i) The sequences were filtered based on their “pintail” values (<95) from the Silva database and then were checked for “chimeric” regions based on the “Gold” reference sequences obtained and implemented through the UCHIIME pipeline (49). (ii) Then the longer sequences (>1,400 bp) were clustered at 99% sequence identity using UCLUST (50). (iii) These representative sequences (246 in total) along with 16S rRNA gene sequences from all available Asgard genomes, including this study (2, 3, 12, 13) together with longer Asgard 16S rRNA gene sequences from a primer-free method (16) (34 in total), were aligned using MAFFT (L-INS-i) (51) against a reference alignment from many representatives throughout TACK and *Bathyarchaeota* phylogeny (65 in total) (see Table S1 posted at figshare [https://doi.org/10.6084/m9.figshare.9257885]). (iv) The alignment was trimmed using Trim-Al operated under the “-gappyout” configuration (52), from which the maximum likelihood (ML) phylogenetic tree was calculated using IQ-TREE (53) with a minimum of 1,000 iterations (-bb 1000 -alrt 1000) and GTR-I-G4 as the best model. (v) Then all other high-quality shorter sequences (pintail value of >95, length of <1,400 bp) were mapped to the reference sequences of the backbone tree using USEARCH (-usearch_local -id 0.95 -evalue 1e−06) (50). (vi) Finally, all the taxonomic affiliations of sequences that differed in the SILVA database from the taxonomic affiliation based on this refined phylogeny (Fig. 1A) were corrected. An in-house script was then used to obtain the respective GenBank file for each of these sequences from the NCBI database (54). From here, the isolation source (environment), pH, and other linked information were obtained mainly based on the original publications that reported these sequences.

### Sample collection.

Two 20-cm sediment cores (cores 1 and 2) were collected at a dry lagoon near Puertecitos on the Baja California Peninsula (latitude, 30°12′37.27″N; longitude, 114°39′48.36″W) in May 2016. Immediately after sampling, the cores were divided into four subsamples of ca. 2 g material collected at 5-cm intervals (1, 5, 10, and 15 cm) and were stored in RNAlater solution (Sigma-Aldrich, Vienna, Austria). Upon arrival in the laboratory (University of Vienna), the samples were washed with phosphate-buffered saline (PBS) buffer to remove the RNAlater solution. DNA for metagenome sequencing was extracted from the top 1-cm horizon of the core with 0.5 g starting material. The protocol included bead beating and phenol-chloroform extraction with 5% cetyltrimethylammonium bromide (CTAB)–phosphate extraction buffer. A second preparation included an additional pretreatment with lysozyme (1.5 mg/ml at 37°C for 30 min; Sigma-Aldrich, Vienna, Austria). Sequences were generated from both DNA preparations to allow for differential coverage binning. Whole-metagenome shotgun sequencing was performed on an Illumina HiSeq2500 (paired-end 150-bp reads) at the Vienna Biocenter Core Facilities (VBCF). Subsamples were taken from both cores for in-depth analysis of the prokaryotic community throughout the sediment horizons, including 16S rRNA gene amplicon sequencing and lipid biomarker analysis, and can be found elsewhere (13, 15).

### Binning.

The raw sequences were trimmed (5-bp window with an average quality value of <20), and the sequencing adaptors were removed using TRIMMOMATIC (55) before the sequences with average low quality (<25) and low complexity were filtered using PRINSEQ (56). Trimmed reads from both lysozyme-treated and untreated samples were coassembled using both metaSPAdes (57) and MEGAHIT (58). The trimmed reads from each of the samples were then mapped to the coassembly using BBMap (59), and the average coverage for each scaffold in each sample was calculated further. The taxonomy of each scaffold was then predicted based on their universally conserved marker proteins (60) from each scaffold matching to the NCBI protein database (NR) as explained for multimetagenome binning strategies (61, 62). The differential coverage binning targeting the assembled scaffolds/contigs of the Asgard archaea was established using the “mmgenome” toolbox (62) from the assembled metagenomes resulting from the two different assembly programs. The reads mapping to the Asgard archaea from these assemblies were then extracted and reassembled together using metaSPAdes. The Asgard archaeal bins (metagenome assembled genomes [MAGs]) were then checked for completeness and contamination using checkM (63).

### Genome annotation and orthologous groups.

The genes from the MAGs were predicted using Prodigal (64). The MAGs in this study were also analyzed with the support of MaGe (65) for various genomic features, including synteny. The COG and arCOG annotations of the proteins in the MAGs were predicted using COGsoft (45, 66). The proteins were also annotated for functions using KEGG (BlastKOALA) (67), BLASTP against nonredundant proteins, and the RefSeq protein database from NCBI (E value of <1e−10). The subcellular localization and the transmembrane domains of the proteins were predicted using multiple tools, including PSORTb, Phobius, and PRED-SIGNAL (68–70). The 16S and 23S rRNA regions in the MAGs were also predicted using RNAmmer (71). The predicted 62 ribosomal proteins (see Table S2 posted at figshare [https://doi.org/10.6084/m9.figshare.9258521]) from the *Loki*- and *Thorarchaeota* were aligned together with the representative organisms from TACK (*Bathyarchaeota* SMTZ-80, Nitrososphaera viennensis and Sulfolobus islandicus LS215), *Euryarchaeota* (Thermoplasma volcanicum GSS1, Halobaculum gomorrense, and Methanothermococcus okinawensis), and DPANN (*Pacearchaeota* RBG-13-36-9 and *Woesearchaeota* UBA94) with MAFFT (L-INS-i). The alignments were then refined with BMGE (72) and concatenated. Further, the phylogeny of the MAGs from this concatenated alignment was inferred using IQ-TREE (ML) with a minimum of 1,000 iterations (-bb 1000 -alrt 1000) and LG+F+I+G4 as the best model. The orthologous group analyses of the MAGs in this study were carried out along with all available genomes from *Loki*- and *Thorarchaeota* (April 2018) in the NCBI database together with the genomes from this study using OrthoFinder (20) (see Table S3 posted at figshare [https://doi.org/10.6084/m9.figshare.9258926]).

### Analysis of putative reductive dehalogenase genes.

The amino acid sequences of the putative Asgard RDases were queried against the EggNOG database (28) with a likelihood threshold of 1e−40. Representative sequences of the resulting orthologous groups were collected from the UniProt database (The UniProt Consortium, 2019) via their accession numbers. Epoxyqueosine reductase (QueG), which shares sequence similarity with RDases (25), was selected as an outgroup for phylogenetic reconstruction. All QueG protein sequences were collected from the KEGG database orthology group for QueG (K18979), and a representative set was generated by application of CD-hit (73) at 50% similarity clustering. The putative Asgard RDases, EggNOG orthogroup representatives, and QueG representatives were aligned with MAFFT –auto (51). The alignment was trimmed using TrimAl –gappyout (52), and subjected to maximum likelihood phylogenetic reconstruction using RaxML rapid bootstrap analysis under the PROTGAMMALG substitution model with 500 iterations. The resulting phylogenetic tree was visualized using the Interactive Tree of Life (74).

Conserved protein domains of the putative Asgard RDases were compared to those of RDases with demonstrated reductive dechlorination activity as described previously (75) and the putative RDase of Ferroglobus placidus. pfam domains and signal peptides were characterized using the NCBI Web CD-Search Tool (76) and SignalP 5.0 (77), respectively. A maximum likelihood phylogenetic tree was constructed using the archaeal and proven-function RDases using MAFFT, TrimAl, RaxML, and the Interactive Tree of Life as described above.

BLASTp (78) with an E value threshold of 1e−10 was used to perform pairwise comparisons between protein sets encoded in putative RDase gene neighborhoods. Thorarchaeotal synteny was characterized by comparing all coding regions found on the RDase-containing scaffolds of Thor_SMTZ1-83, Thor_AB, and Thor_SMTZ1-45 to the 15 downstream and 26 upstream coding regions relative to the RDase detected in Baja_Thor. For *Lokiarchaeota*, sequential pairwise comparisons between all coding regions on the RDase-containing scaffolds were performed as shown in Fig. S5. BLASTp alignment results were visualized using the genoPlotR package (79). KEGG orthology annotations were assigned using GhostKoala (80), and COG and TIGRFAM annotations were assigned using the WebMGA server with E value thresholds of 1e−3. The protein sequences in MAGs predicted to be involved in the pathways of anaerobic degradation of aromatic amino acids were inferred through the pathways in Ferroglobus placidus (33) (BLASTp [>35% amino acid identity and >85% query coverage]).

### Annotation of lipid biosynthesis pathways.

The different enzymes involved in the lipid biosynthesis pathway(s) identified in all MAGs from *Loki*- and *Thorarchaeota* were predicted based on the different functional annotation tools, including HMMER3 and orthogroup analysis. These enzymes were inferred based on the known enzymes and pathways (40). The phylogeny of the predicted enzymes such as glyceraldehyde-3-phosphate dehydrogenase (*glpAD*) and glycerol kinase (*glpK*) were obtained together with those of the other enzymes involved in the process (40). Similarly, the phylogeny of glyceraldehyde-1-phosphate dehydrogenases (G1PDH) in *Lokiarchaeota* was predicted together with those of different members of the glycerol dehydrogenase superfamily (*glp* and *gps*) and also with manually curated G1PDHs from UniProt. The Lokiarchaeal G1PDHs were aligned specifically with the bacterial G1PDHs (Bacillus subtilis) using MAFFT (L-INS-i) to understand their relationship (41).

### Data availability.

The genome bins from this study can be found at NCBI BioProject ID PRJNA521734. The MAGs from this study have been deposited under the following accession numbers: Baja_Thor (“*Candidatus* Thorarchaeota archaeon” BC), SHMX00000000; Baja_Loki1 (“*Candidatus* Lokiarchaeota archaeon” BC1), SHMU00000000; Baja_Loki2 (“*Candidatus* Lokiarchaeota archaeon” BC2), SHMV00000000; and Baja_Loki3 (“*Candidatus* Lokiarchaeota archaeon” BC3), SHMW00000000.
