# Erratum for Manoharan et al., “Metagenomes from Coastal Marine Sediments Give Insights into the Ecological Role and Cellular Features of *Loki*- and *Thorarchaeota*”

**DOI:** 10.1128/mBio.02509-19

**Published:** 2019-10-29

**Authors:** Lokeshwaran Manoharan, Jessica A. Kozlowski, Robert W. Murdoch, Frank E. Löfﬂer, Filipa L. Sousa, Christa Schleper

**Affiliations:** a Archaea Biology and Ecogenomics Division, University of Vienna, Vienna, Austria; b Center for Environmental Biotechnology, University of Tennessee, Knoxville, Tennessee, USA; c Department of Microbiology, Department of Civil and Environmental Engineering, and Department of Biosystems Engineering and Soil Science, University of Tennessee, Knoxville, Tennessee, USA; d Biosciences Division, Oak Ridge National Laboratory, Oak Ridge, Tennessee, USA

## ERRATUM

Volume 10, no. 5, e02039-19, 2019, https://doi.org/10.1128/mBio.02039-19. The affiliation of coauthor Frank E. Löffler with Oak Ridge National Laboratory was inadvertently omitted. The corrected author affiliations are shown above.

