# Supplementary figures and images for: Metagenomes from Coastal Marine Sediments Give Insights into the Ecological Role and Cellular Features of Loki- and Thorarchaeota
Source: mBio. 2019 Sep 10;10(5):e02039-19. doi: 10.1128/mBio.02039-19 (PMC6737245; doi:10.1128/mBio.02039-19)

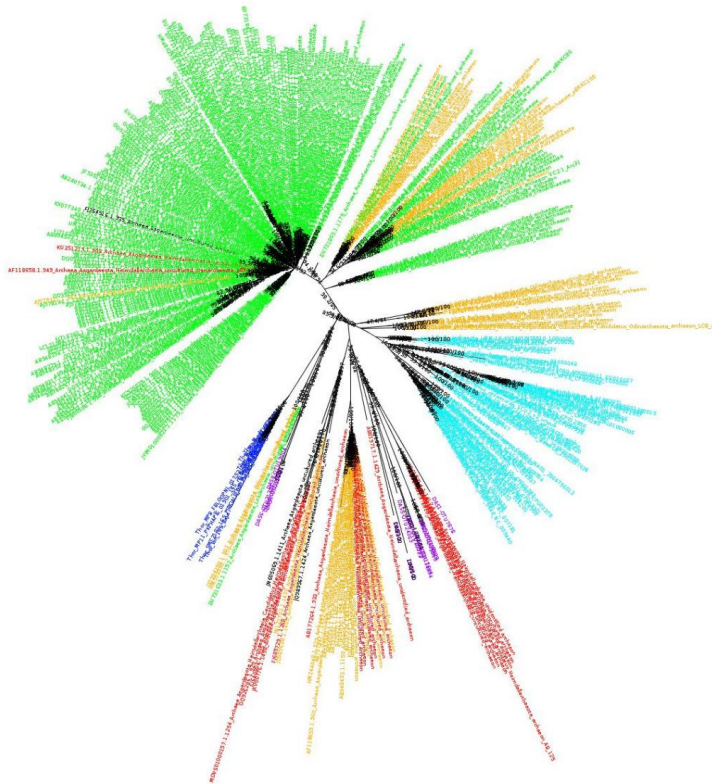

- Lokiarchaea
- Thorarchaea
- Odinarchaea
- Heimdallarchaea
- DAS
- Unknown Asgard
- TACK

Supplement: FIG S1 [file mBio.02039-19-sf001.pdf]

Tree scale: 0.1

Colored ranges

- Odin
- Heim
- DAS
- Thor
- Loki-A
- Loki-B1
- Loki-B2
- Loki-C

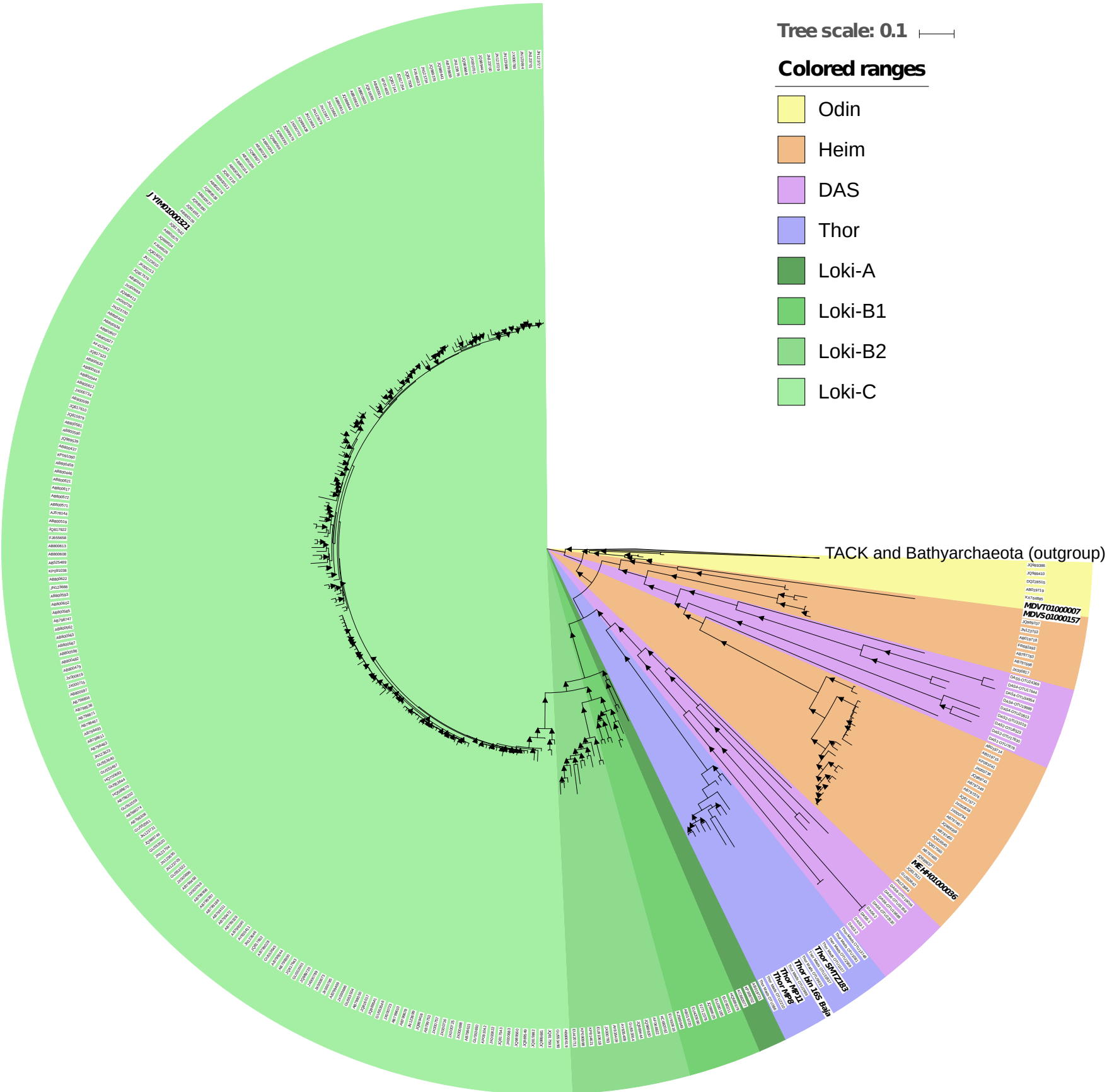

Supplement: FIG S2 [file mBio.02039-19-sf002.pdf]

# A) Lokiarchaeota

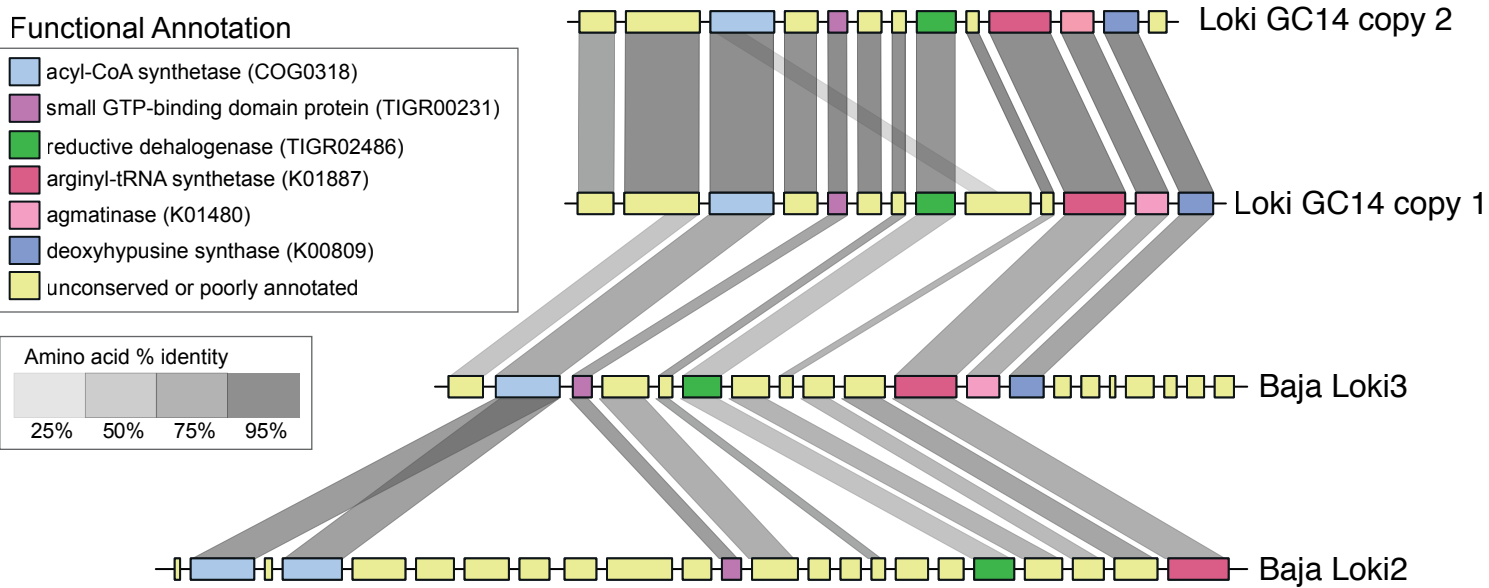

# B) Thorarchaeota

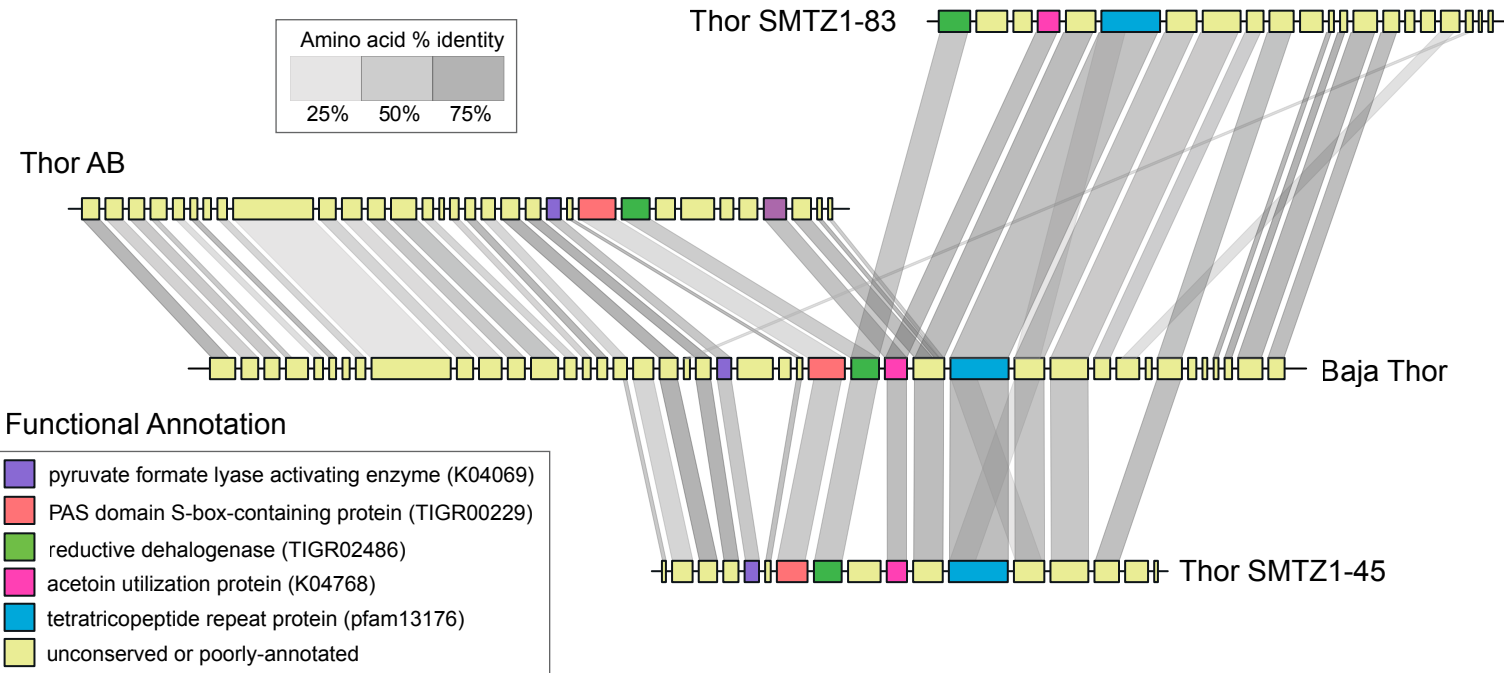

Supplement: FIG S5 [file mBio.02039-19-sf005.pdf]

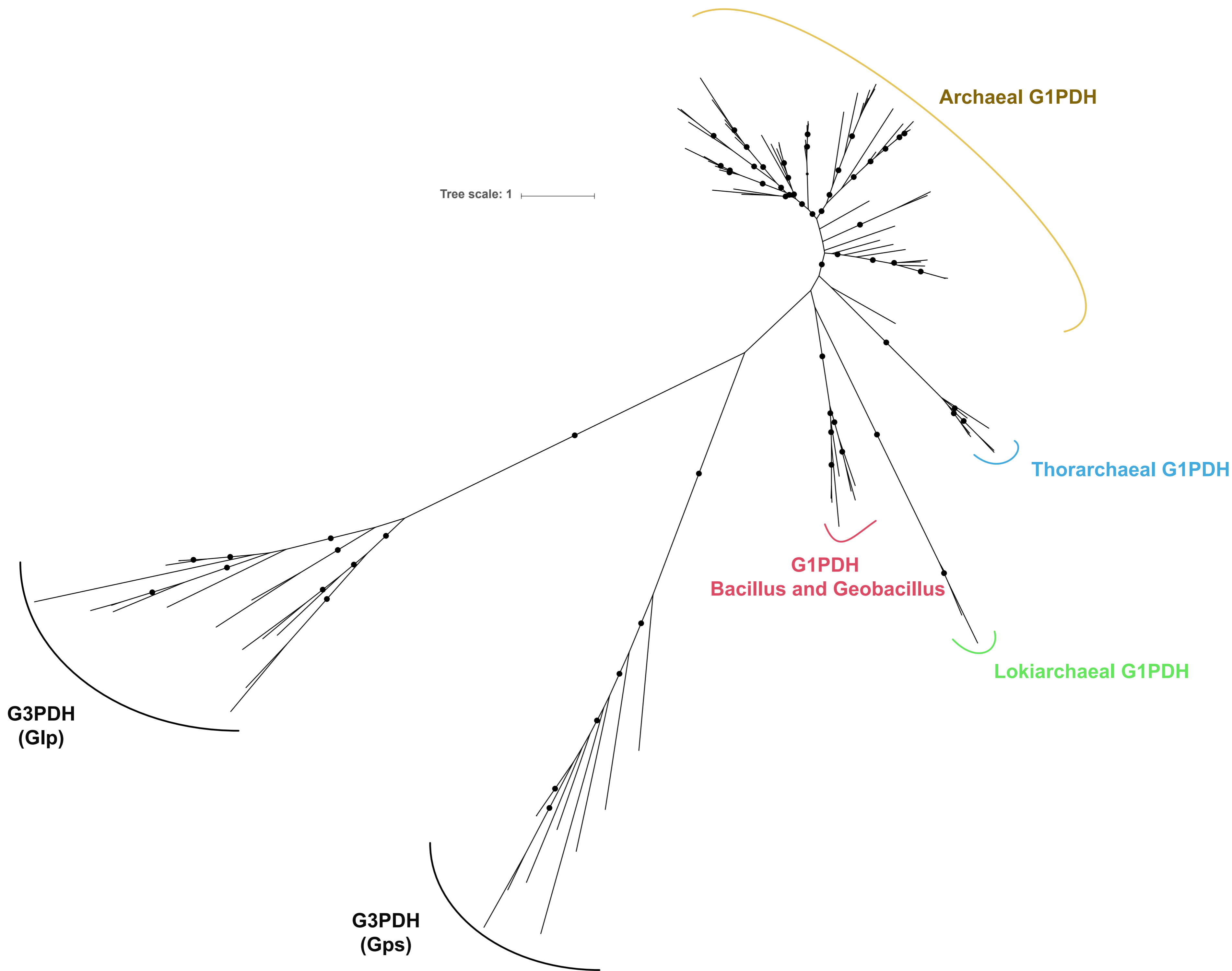

Supplement: FIG S7 [file mBio.02039-19-sf007.pdf]

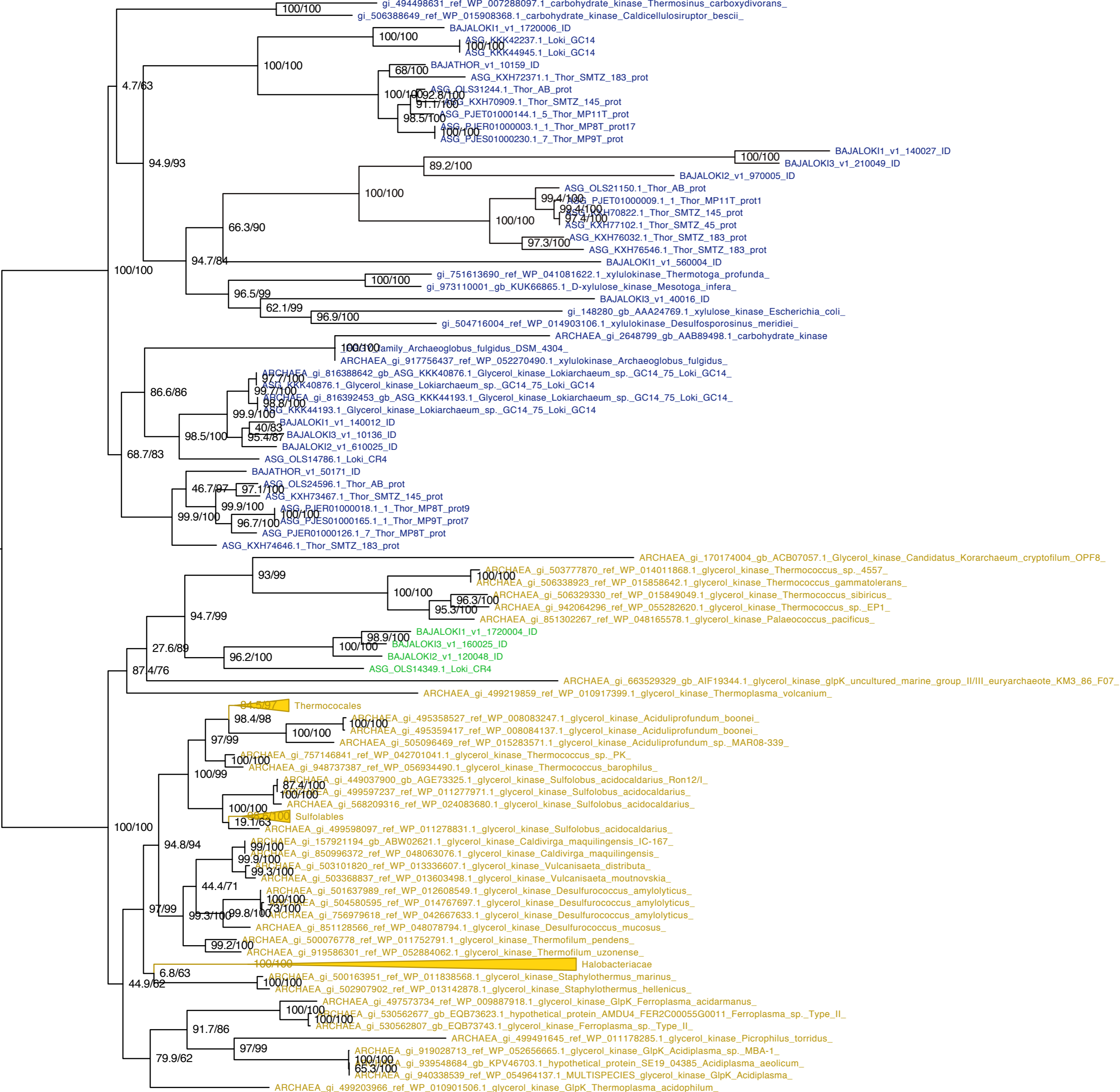

Supplement: FIG S8 [file mBio.02039-19-sf008.pdf]
