# Supplementary material for: Metagenomes from Coastal Marine Sediments Give Insights into the Ecological Role and Cellular Features of Loki- and Thorarchaeota
Source: mBio. 2019 Sep 10;10(5):e02039-19. doi: 10.1128/mBio.02039-19 (PMC6737245; doi:10.1128/mBio.02039-19)
Supplement: FIG S3 [file mBio.02039-19-sf003.pdf]

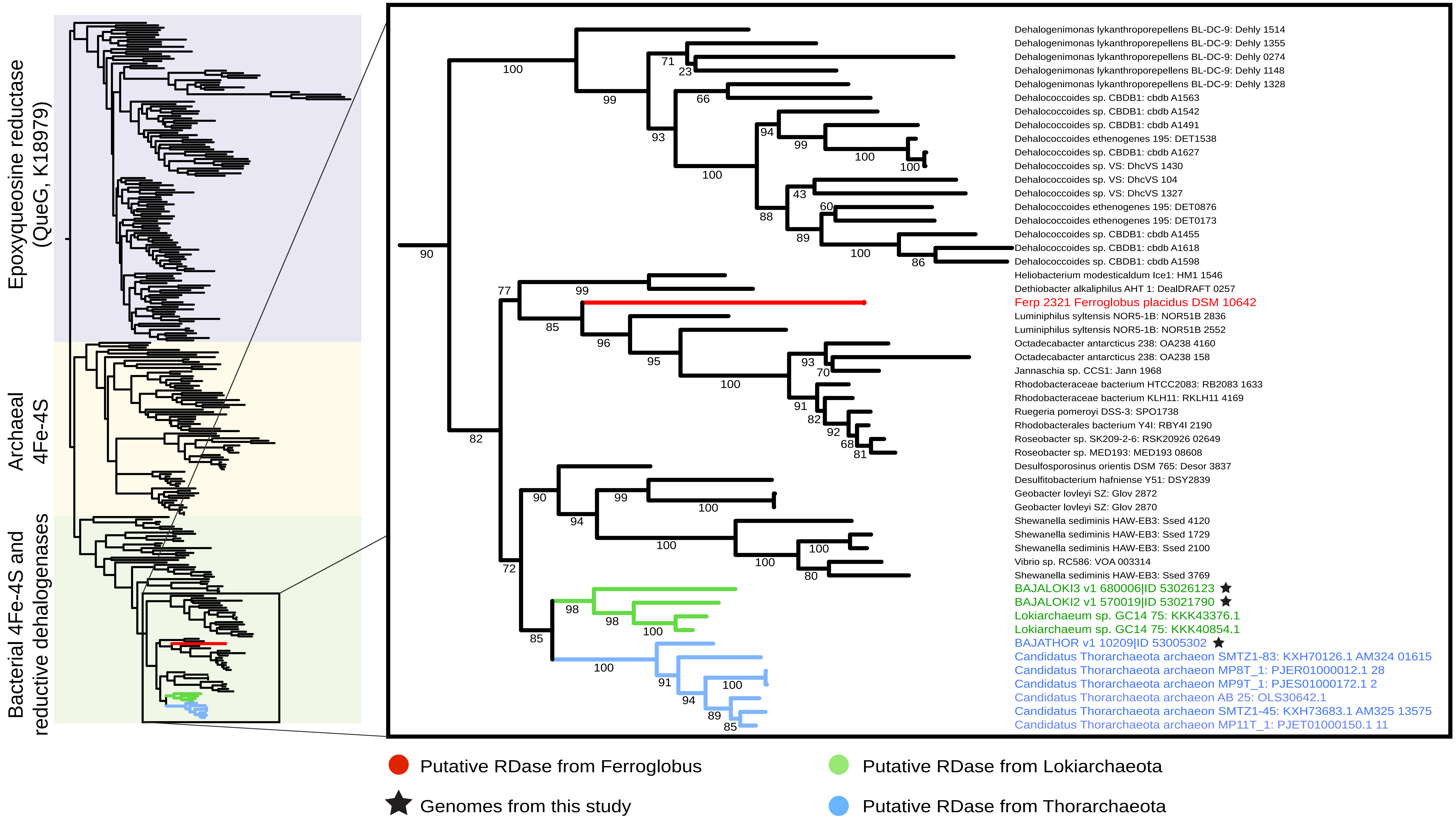
Epoxyqueosine reductase (QueG, K18979)
Archaeal 4Fe-4S
Bacterial 4Fe-4S and reductive dehalogenases
Dehalogenimonas lykanthroporepellens BL-DC-9: Dehly 1514
Dehalogenimonas lykanthroporepellens BL-DC-9: Dehly 1355
Dehalogenimonas lykanthroporepellens BL-DC-9: Dehly 0274
Dehalogenimonas lykanthroporepellens BL-DC-9: Dehly 1148
Dehalogenimonas lykanthroporepellens BL-DC-9: Dehly 1328
Dehalococcoides sp. CBDB1: cbdb A1563
Dehalococcoides sp. CBDB1: cbdb A1542
Dehalococcoides sp. CBDB1: cbdb A1491
Dehalococcoides ethenogenes 195: DET1538
Dehalococcoides sp. CBDB1: cbdb A1627
Dehalococcoides sp. VS: DhcVS 1430
Dehalococcoides sp. VS: DhcVS 104
Dehalococcoides sp. VS: DhcVS 1327
Dehalococcoides ethenogenes 195: DET0876
Dehalococcoides ethenogenes 195: DET0173
Dehalococcoides sp. CBDB1: cbdb A1455
Dehalococcoides sp. CBDB1: cbdb A1618
Dehalococcoides sp. CBDB1: cbdb A1598
Heliobacterium modesticaldum Ice1: HM1 1546
Dethiobacter alkaliphilus AHT 1: DealDRAFT 0257
Ferp 2321 Ferroglobus placidus DSM 10642
Luminiphilus syltensis NOR5-1B: NOR51B 2836
Luminiphilus syltensis NOR5-1B: NOR51B 2552
Octadecabacter antarcticus 238: OA238 4160
Octadecabacter antarcticus 238: OA238 158
Jannaschia sp. CCS1: Jann 1968
Rhodobacteraceae bacterium HTCC2083: RB2083 1633
Rhodobacteraceae bacterium KLH11: RKLH11 4169
Ruegeria pomeroyi DSS-3: SPO1738
Rhodobacterales bacterium Y4I: RBY4I 2190
Roseobacter sp. SK209-2-6: RSK20926 02649
Roseobacter sp. MED193: MED193 08608
Desulfosporosinus orientis DSM 765: Desor 3837
Desulfitobacterium hafniense Y51: DSY2839
Geobacter lovleyi SZ: Glov 2872
Geobacter lovleyi SZ: Glov 2870
Shewanella sediminis HAW-EB3: Ssed 4120
Shewanella sediminis HAW-EB3: Ssed 1729
Shewanella sediminis HAW-EB3: Ssed 2100
Vibrio sp. RC586: VOA 003314
Shewanella sediminis HAW-EB3: Ssed 3769
BAJALOKI3 v1 680006|ID 53026123 ★
BAJALOKI2 v1 570019|ID 53021790 ★
Lokiarchaeum sp. GC14 75: KKK43376.1
Lokiarchaeum sp. GC14 75: KKK40854.1
BAJATHOR v1 10209|ID 53005302 ★
Candidatus Thorarchaeota archaeon SMTZ1-83: KXH70126.1 AM324 01615
Candidatus Thorarchaeota archaeon MP8T_1: PJER01000012.1 28
Candidatus Thorarchaeota archaeon MP9T_1: PJES01000172.1 2
Candidatus Thorarchaeota archaeon AB 25: OLS30642.1
Candidatus Thorarchaeota archaeon SMTZ1-45: KXH73683.1 AM325 13575
Candidatus Thorarchaeota archaeon MP11T_1: PJET01000150.1 11
Putative RDase from Ferroglobus
Putative RDase from Lokiarchaeota
Genomes from this study
Putative RDase from Thorarchaeota
