# Supplementary material for: Metagenomes from Coastal Marine Sediments Give Insights into the Ecological Role and Cellular Features of Loki- and Thorarchaeota
Source: mBio. 2019 Sep 10;10(5):e02039-19. doi: 10.1128/mBio.02039-19 (PMC6737245; doi:10.1128/mBio.02039-19)
Supplement: FIG S6 [file mBio.02039-19-sf006.pdf]

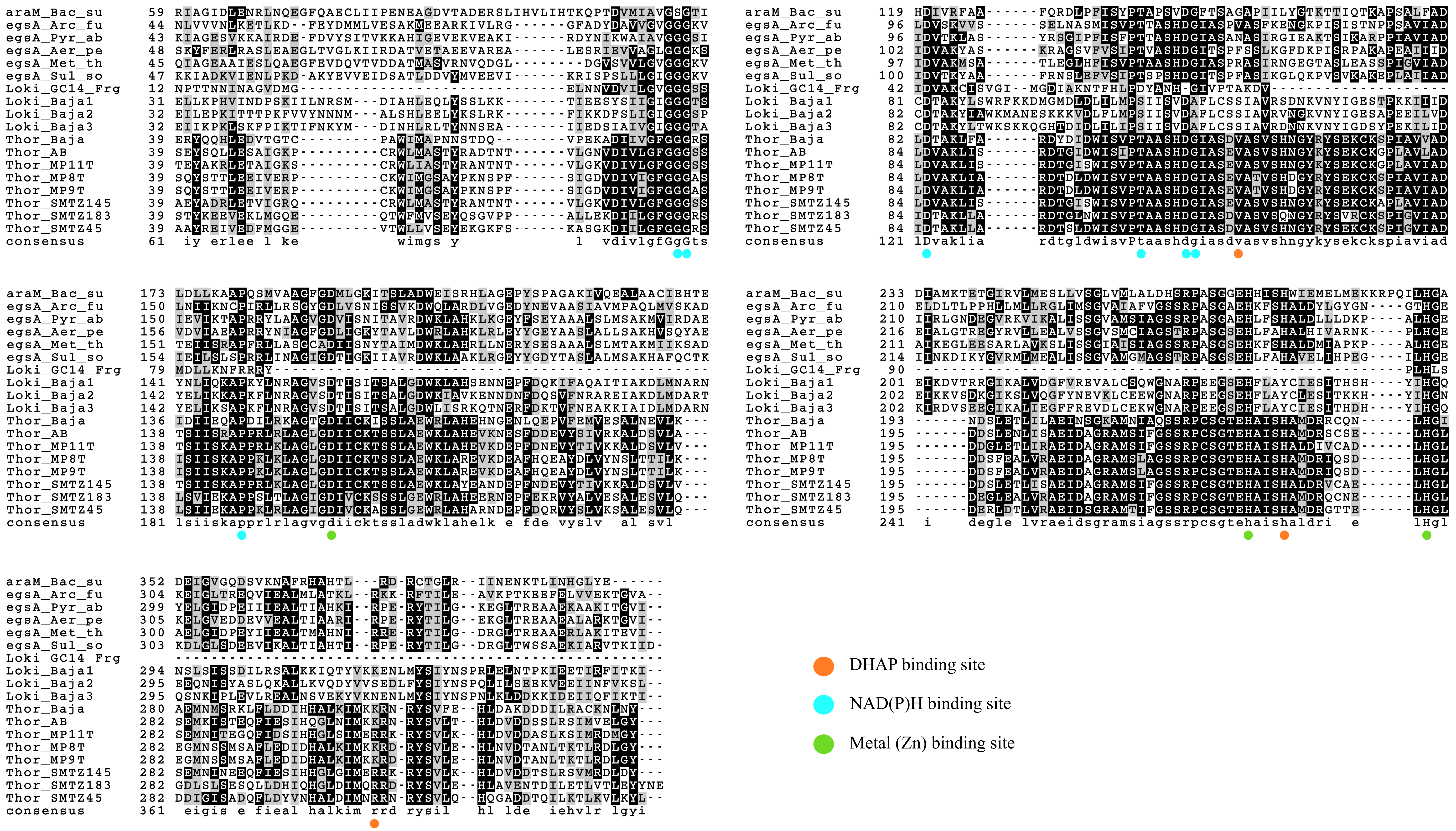

DHAP binding site
NAD(P)H binding site
Metal (Zn) binding site
