# Supplementary material for: Metagenomes from Coastal Marine Sediments Give Insights into the Ecological Role and Cellular Features of Loki- and Thorarchaeota
Source: mBio. 2019 Sep 10;10(5):e02039-19. doi: 10.1128/mBio.02039-19 (PMC6737245; doi:10.1128/mBio.02039-19)
Supplement: FIG S4 [file mBio.02039-19-sf004.pdf]

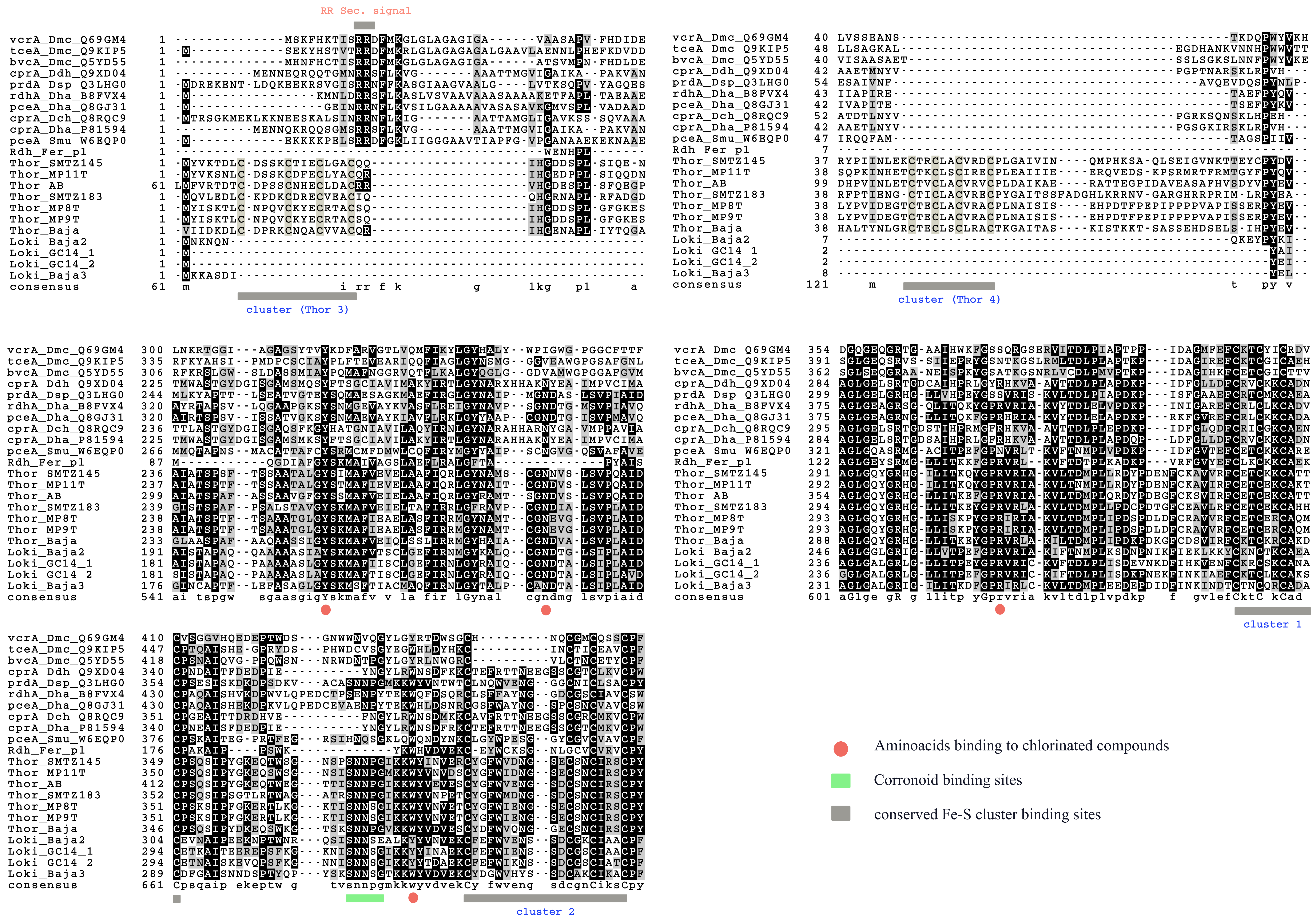
RR Sec. signal
cluster (Thor 3)
cluster (Thor 4)
cluster 1
cluster 2
Aminoacids binding to chlorinated compounds
Corronoid binding sites
conserved Fe-S cluster binding sites
